# Combined blood purification and antiarrhythmic therapy for acute aconitine poisoning with refractory arrhythmias: a case-based mechanistic evaluation and treatment strategy optimization

**DOI:** 10.1186/s40001-025-03167-1

**Published:** 2025-09-29

**Authors:** Cheng Yang, Ting Zou, Qian-Hui Zang, Wei Cao, Yuan Suo

**Affiliations:** 1 Emergency Department, The First Hospital of Jiaxing/The Affiliated Hospital of Jiaxing University, No. 1882 Zhonghuan South Road, Nanhu District, Jiaxing, 314000 Zhejiang China; 2 Infectious Diseases Department, The First Hospital of Jiaxing, The Affiliated Hospital of Jiaxing University, Jiaxing, 314000 China; 3 Emergency Department, Dangtu People’s Hospital, Maanshan 243100, Anhui, China

**Keywords:** Aconitine poisoning, Arrhythmia, Hypotension, Blood purification, Electrical cardioversion, Comprehensive treatment strategy

## Abstract

**Introduction:**

Aconitine poisoning from traditional Chinese medicine is life-threatening, associated with arrhythmias and shock. Early diagnosis and multidisciplinary treatment are essential due to the lack of specific antidotes. This study aimed to present a severe case of aconitine poisoning and to evaluate the effectiveness of combined blood purification and antiarrhythmic therapy, thereby providing practical insights for clinical management.

**Case summary:**

A 70-year-old man presented with coma, recurrent ventricular arrhythmias, and severe hypotension (nadir: 44/24 mmHg) after ingesting Fuzhi (Aconitum taipeicum). Toxicology confirmed high aconitine levels. He was treated with norepinephrine (0.17–0.33 μg/kg/min), intravenous amiodarone (150 mg bolus over 10–15 min, then 1 mg/min infusion), electrical cardioversion, and early blood purification (hemoperfusion + CVVH). He regained hemodynamic stability within 24 h and fully recovered at 6 months (LVEF 59–63%).

**Conclusions:**

Combined antiarrhythmic therapy and blood purification proved effective. Early recognition and integrated care are key to managing aconitine poisoning.

**Graphical abstract:**

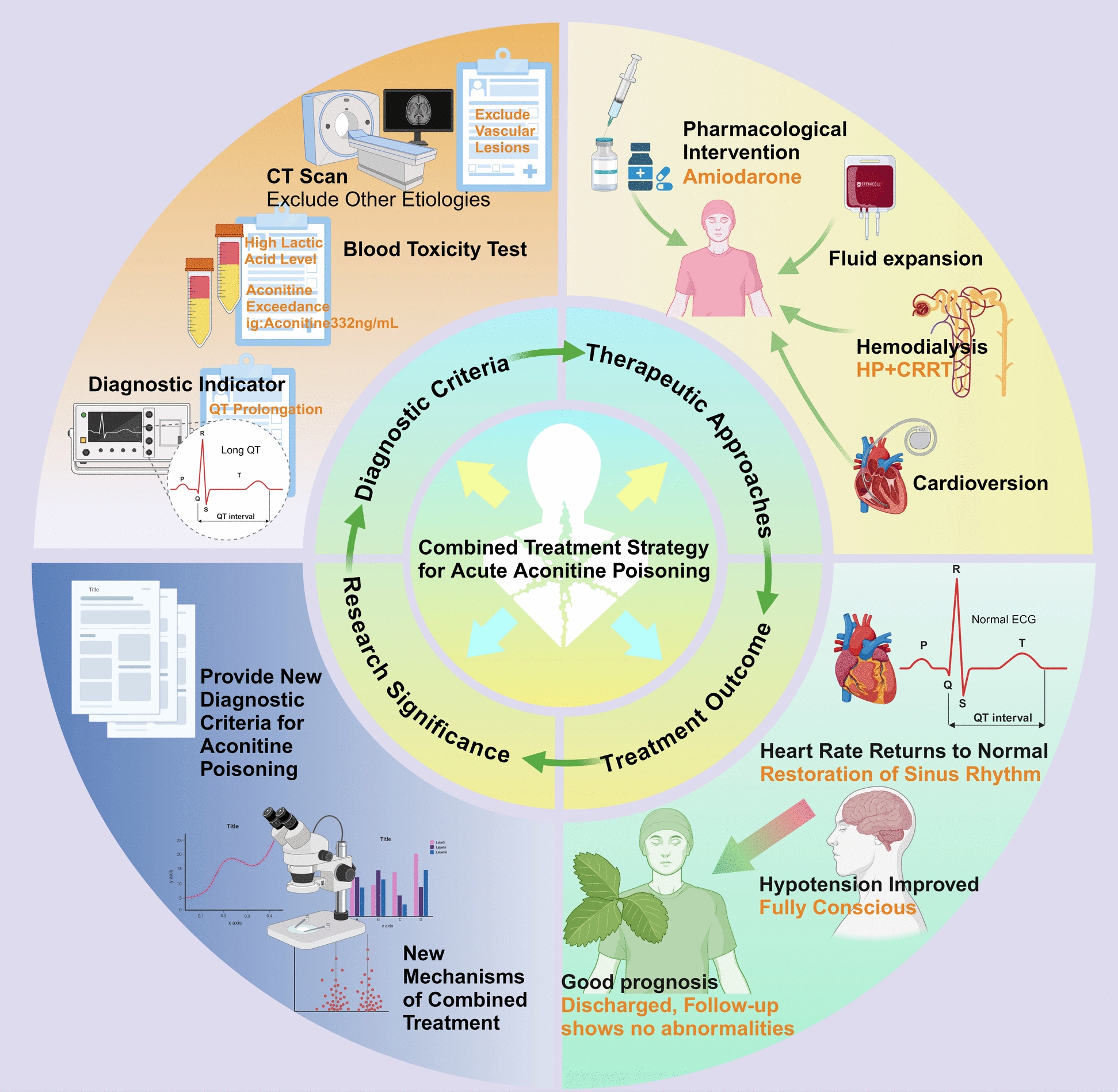

## Introduction

Aconite species, such as Chuanwu (*Aconitum carmichaelii*), Caowu (*Aconitum kusnezoffii*), and Fuzhi (*Aconitum taipeicum*), are widely used in traditional Chinese medicine due to their significant pharmacological effects [1]. The alkaloids in these plants exhibit a range of therapeutic properties, including anti-inflammatory, analgesic, cardiotonic, and anticancer activities, and are commonly used to treat rheumatic diseases, cardiovascular disorders, and certain types of cancer [2]. Particularly in traditional Chinese medicine formulations, Chuanwu and Fuzhi are highly regarded for their synergistic effects in enhancing therapeutic efficacy. However, the toxicity of these substances is substantial [3, 4], with a narrow therapeutic-to-toxic dose range. Even slight mismanagement can result in severe adverse effects.

The toxic mechanism of aconite alkaloids primarily involves the prolonged activation of voltage-gated sodium channels, which leads to sustained cellular depolarization at the plateau level of the action potential. This process disrupts the normal functioning of myocardial cells and the nervous system, manifesting as severe arrhythmias, hypotension, and central nervous system (CNS) depression [5]. Research indicates that the oral dose of aconitine required to induce poisoning is as low as 0.2 mg, with doses exceeding 2–4 mg potentially being fatal [6]. The extremely narrow dose range significantly increases the clinical risks associated with the use of aconite-based medications, particularly when the plant is improperly processed or used incorrectly, making severe poisoning more likely.

In recent years, both isolated and mass-poisoning incidents involving aconite have been frequently reported worldwide [7]. This trend is related to the widespread use of aconite in traditional Chinese medicine and reflects the public's insufficient awareness of its toxicity [8]. Common causes of poisoning include the ingestion of improperly processed aconite herbs, homemade alcoholic tinctures containing aconitine, and improper use of compound traditional medicines containing aconite alkaloids [8, 9]. Acute aconite poisoning presents diverse clinical manifestations, primarily involving acute cardiovascular damage, which typically includes symptoms such as nausea, vomiting, hypotension, ventricular arrhythmias, and CNS depression [9]. Severe cases may progress to circulatory failure and cardiac arrest, resulting in a high mortality rate [10, 11]. Treatment of poisoning cases generally requires multidisciplinary intervention, but challenges such as delayed diagnosis, limited treatment options, and difficulties in toxin elimination persist.

This report presents a case of acute aconitine poisoning with severe hypotension and coma caused by recurrent ventricular arrhythmias. Through analysis of the clinical course and outcome, combined with a review of relevant literature, this study aims to provide practical guidance for the diagnosis, treatment, and prevention of aconite poisoning and to inform safer clinical use and regulation of aconite-containing medicines.

## Case report

**Fig. 1:**
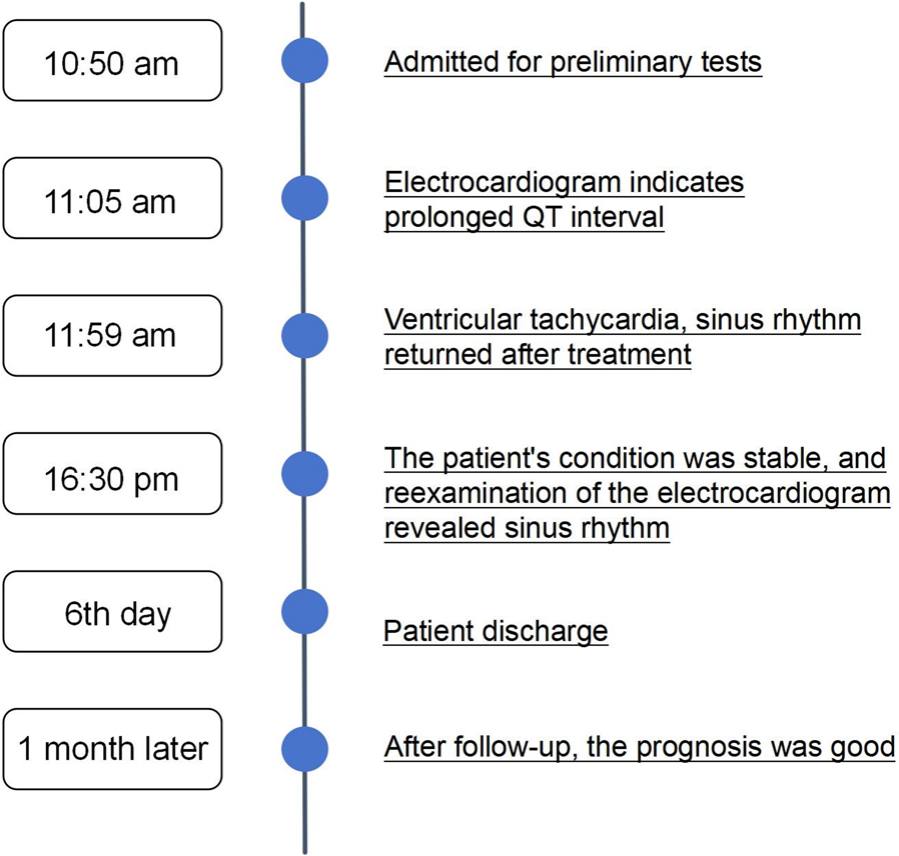
Patient's hospitalization timeline

A 70-year-old male with a history of hypertension was admitted to the emergency department of the First Hospital of Jiaxing, China, at 10:50 A.M., after 1.5 h of unconsciousness (hospital timeline shown in Fig. 1, Table 1). On admission, vital signs were: temperature 36℃, pulse 81 beats/min, respiration 20 breaths/min, and blood pressure 107/80 mmHg. The patient was comatose (GCS E1V2M4, score 7). Physical examination revealed equal and round pupils with sluggish light reflexes, clear bilateral lung sounds, irregular heart rhythm without murmurs, a soft abdomen, and non-cooperation with abdominal and limb strength testing. Pain stimulation elicited no movement in the left limbs, and bilateral Babinski signs were positive.

**Table 1:** Patient timeline

Timepoint	Event
10:50 A.M.	Admission, initial examination
11:05 A.M.	ECG shows prolonged QT interval
11:59 A.M.	Ventricular tachycardia, restored to sinus rhythm after treatment
4:30 P.M.	Condition stabilized, follow-up ECG shows sinus rhythm
Day 6	Patient discharged
1 month later	Follow-up, favorable prognosis

**Fig. 2:**
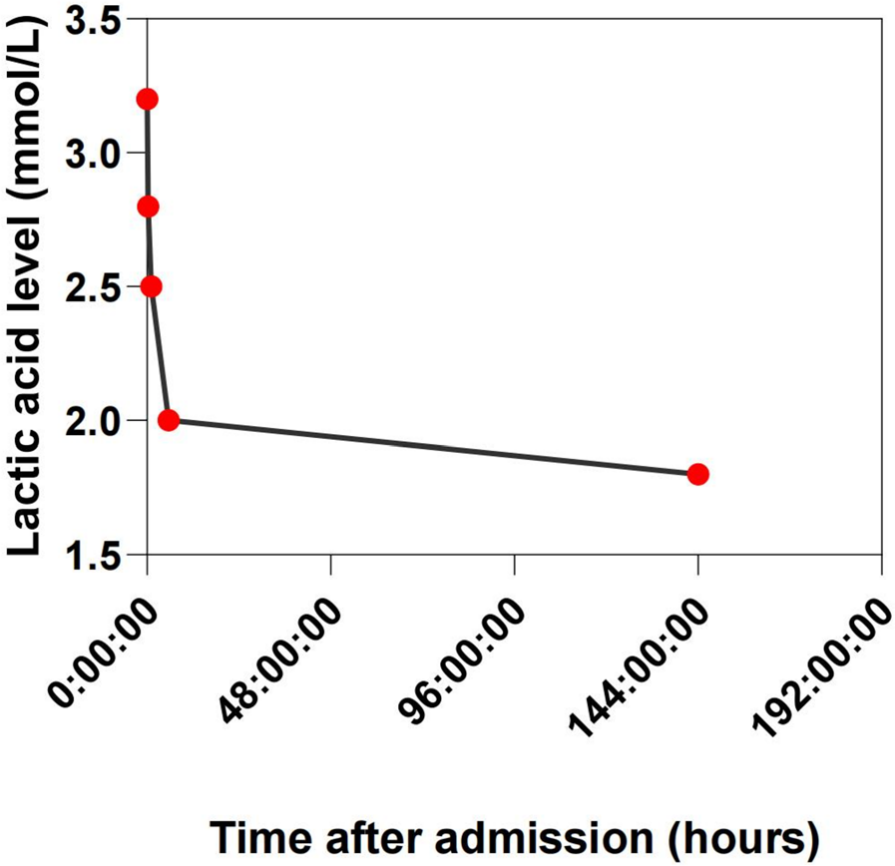
Changes in lactate levels during the patient's treatment

Following admission to the intensive care unit, urgent laboratory testing revealed a blood glucose of 6.3 mmol/L, arterial pH 7.334, base excess (BE) − 8.2 mmol/L, and lactate 3.2 mmol/L (Fig. 2, Table 2). D-dimer was 3,180 ng/mL. Liver, kidney function, and cardiac enzymes (troponin I, creatine kinase) were unremarkable. Cranial CT was normal. Given coma, hemiplegia, and bilateral Babinski signs, ischemic stroke was initially suspected. However, after the CT scan, blood pressure dropped to a nadir of 44/24 mmHg, and consciousness fluctuated. Neurology consultation suggested ischemic stroke was unlikely and prioritized the management of hypotension.

**Table 2:** Lactate level changes during treatment

Timepoint	Lactate level (mmol/L)
Admission (day 1)	3.2
11:05 A.M.	2.8
11:59 A.M.	2.5
4:30 P.M.	2
Discharge (day 6)	1.8

**Fig. 3:**
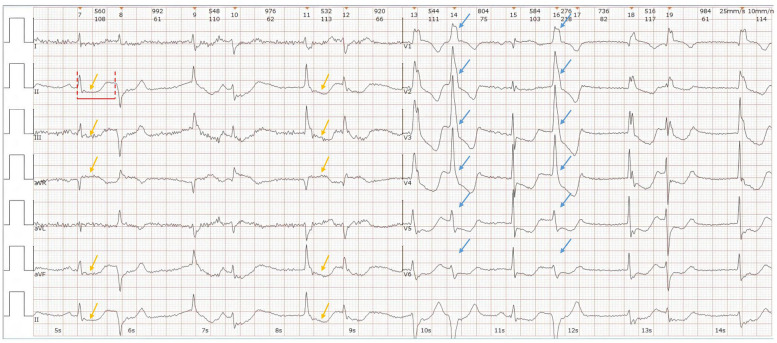
Patient's ECG (11:05 A.M.). (1) Sinus rhythm; (2) multifocal frequent ventricular premature beats, some in couplets and others associated with accelerated ventricular escape rhythm; (3) ST-T segment changes; and (4) prolonged QT interval. Blue arrows indicate ventricular premature beats, yellow arrows indicate ST-T segment changes, and the red line indicates QT interval prolongation

To further investigate, contrast-enhanced CT angiography excluded pulmonary embolism, aortic dissection, and acute myocardial infarction. Echocardiography showed a full left atrium, mild tricuspid regurgitation, and preserved LVEF (59%) with no regional wall motion abnormalities. Electrocardiogram (ECG, Fig. 3) demonstrated sinus rhythm, frequent multifocal premature ventricular contractions (some in couplets, others with accelerated ventricular escape), ST-T changes, and a prolonged QT interval (QT 520 ms, QTc 560 ms). No torsade de pointes arrhythmias were observed during hospitalization.

**Fig. 4:**
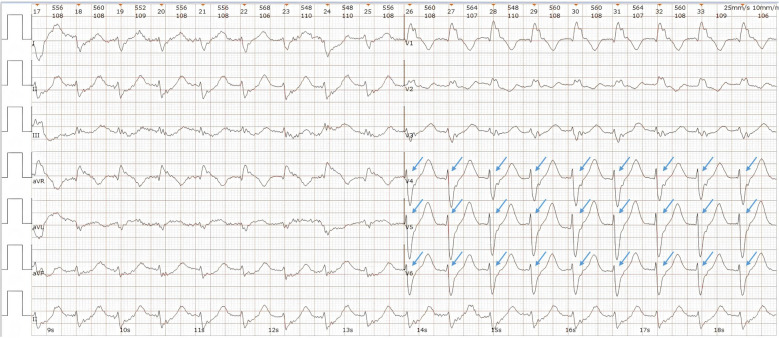
Patient's ECG (11:59 A.M.). The blue arrows indicate ventricular tachycardia

Initial treatment included central venous catheterization, fluid resuscitation, and continuous intravenous norepinephrine infusion (0.17–0.33 μg/kg/min). The patient regained consciousness and limb strength (grade 4); Babinski's signs became negative. ECG at 11:59 A.M. revealed monomorphic ventricular tachycardia at a rate of 180/min (Fig. 4), which spontaneously resolved to sinus rhythm after ~ 20 s. Amiodarone was administered to prevent recurrent ventricular arrhythmias: an intravenous bolus of 150 mg over 10–15 min, then continuous infusion at 1 mg/min for 24 h. The final diagnosis was malignant arrhythmia causing hemodynamic instability and coma.

**Fig. 5:**
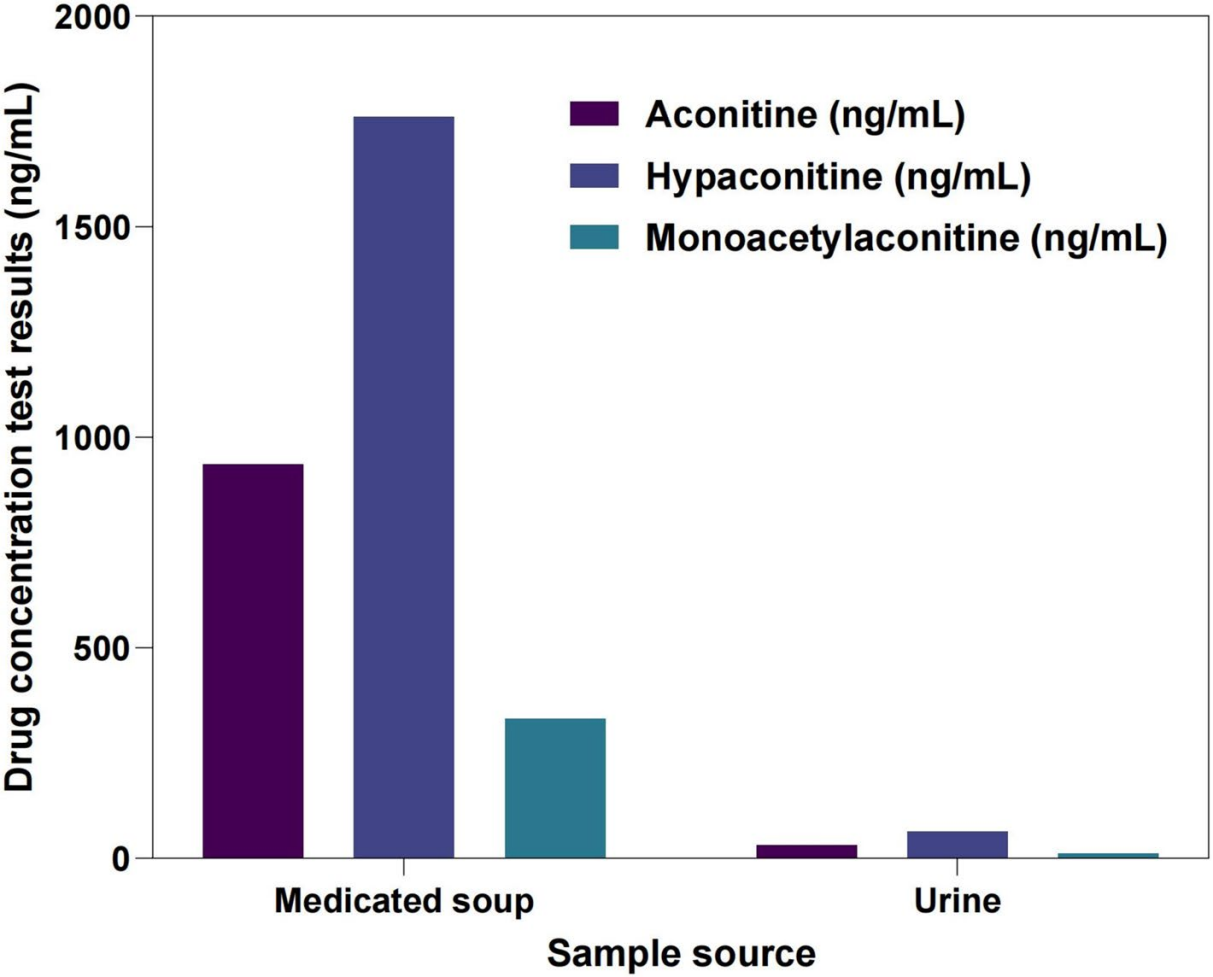
Medication and urine test results

**Fig. 6:**
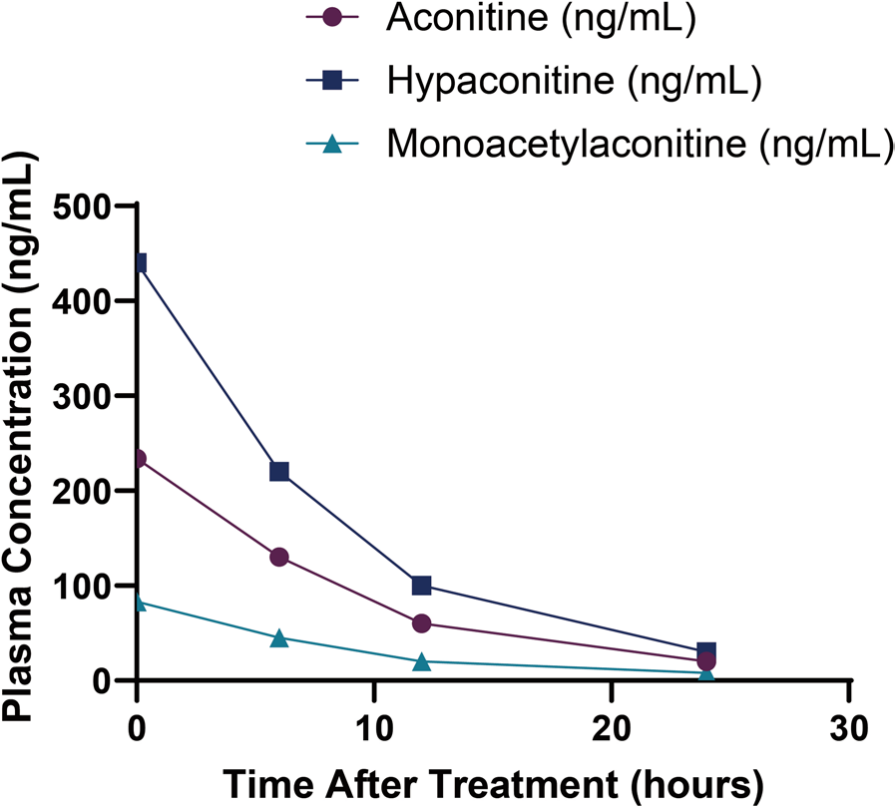
Trends in plasma concentrations of three aconitine alkaloids following combined hemoperfusion (HP) and continuous venovenous hemofiltration (CVVH) therapy

To clarify the etiology, the patient's medical history was further reviewed. The patient had no prior heart disease, cardiac enzymes were normal, and no history of taking antibiotics, antihistamines, antidepressants, antifungal agents, or other QT-prolonging drugs was reported A long history of traditional Chinese medicine use was identified on detailed questioning, including a new formula containing 10 g of Fuzhi (Aconitum) taken 10 days before admission. Fuzhi contains highly toxic diterpenoid alkaloids-aconitine (Fig. 5, Table 3), hypaconitine, and monoacetylaconitine. Toxicological analysis revealed concentrations in the herbal decoction of 332, 936, and 1,760 ng/mL, respectively, all exceeding published toxicity thresholds (> 100, > 100, > 500 ng/mL). Even after 24 h of treatment, urine showed residual toxin (13.3, 32.1, and 65.2 ng/mL), indicating ongoing risk [12]. Combined hemoperfusion (HP) and continuous venovenous hemofiltration (CVVH) were performed, with plasma toxin levels declining steadily within 24 h (Fig. 6), suggesting enhanced clearance. Considering clinical history, toxin concentrations, and simulated pharmacokinetics, the diagnosis of acute Fuzhi (Aconitum) poisoning-induced malignant arrhythmia was confirmed.

**Table 3:** Comparison of drug concentration testing results

Sample source	Pseudoaconitine (ng/mL)	New aconitine (ng/mL)	Aconitine (ng/mL)
Medicinal decoction	936	1760	332
Urine	32.1	65.2	13.3
Reference toxicity concentration	> 100	> 500	> 100

**Fig. 7:**
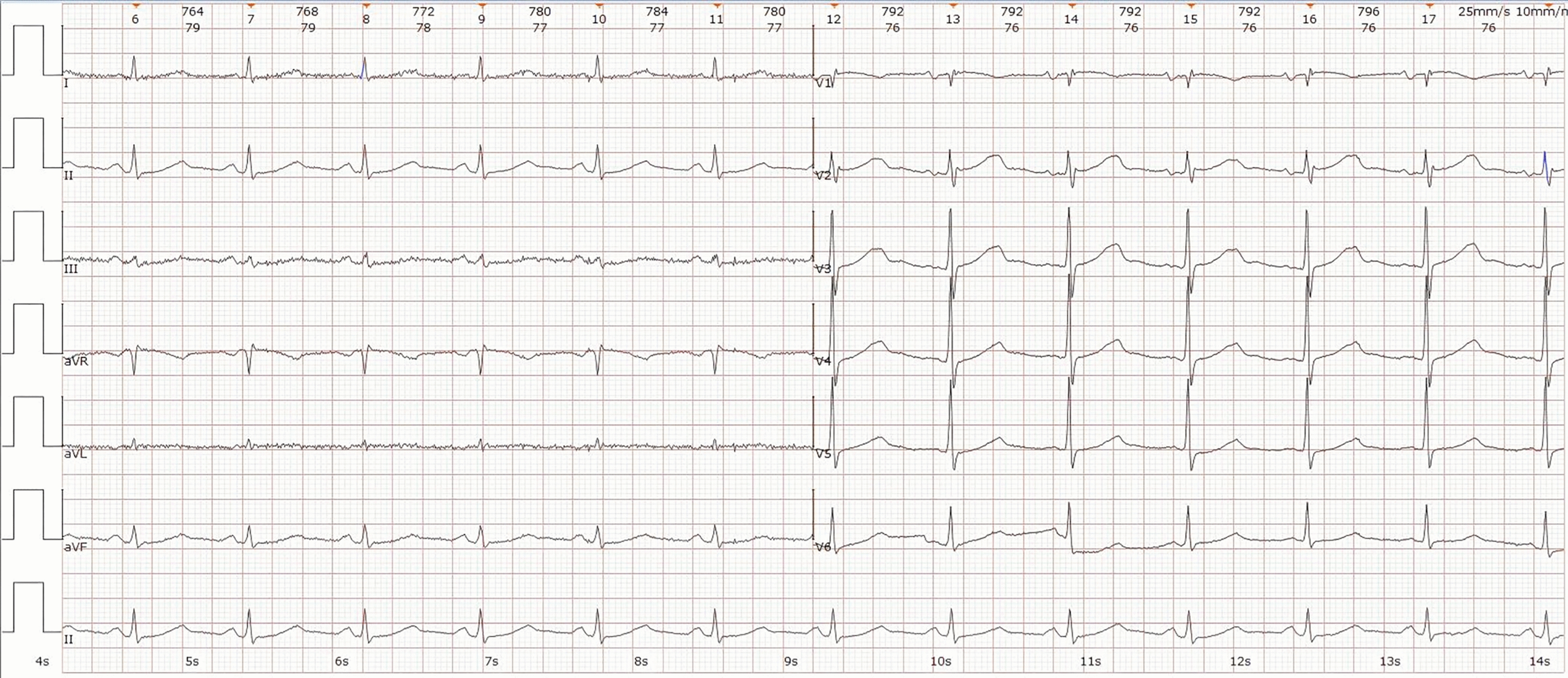
Patient's ECG (4:30 P.M.). Sinus rhythm

The patient was transferred to the emergency intensive care unit (EICU), where follow-up ECG on the day of admission showed sinus rhythm (Fig. 7), with QT interval 480 ms and QTc 552 ms (Fig. 7). His condition stabilized the following day, allowing transfer to a general ward and discharge on day 6.

Long-term follow-up demonstrated favorable recovery. The patient was asymptomatic 1 month post-discharge, with normal ECG and LVEF of 59%. At 3 months, quality of Life improved, with the ability to perform Light work, sinus rhythm maintained, and LVEF increased to 61%. By 6 months, full recovery of daily activity was achieved, dynamic ECG showed no arrhythmias, and LVEF further improved to 63%. The patient continued oral amiodarone with regular blood level monitoring.

## Discussion

Aconitum species, with approximately 350 species globally, are primarily distributed in Asia, followed by Europe and North America [9]. Due to their unique pharmacological properties in traditional Chinese medicine, aconitum plants are widely used in Asia, although less so in Europe and the United States [13, 14]. In China, most aconitum species are found in the Tibetan Plateau and surrounding regions, with fewer in the southeast and Taiwan, leading to regional disparities in clinical experience and prognosis for aconitine poisoning. Multi-system manifestations, including perioral numbness, nausea, vomiting, and chest tightness characterize acute aconitine poisoning. Severe cases may progress to coma, cardiogenic shock, and even death [9]. Mechanistically, aconitine exerts its toxicity mainly through persistent activation of voltage-gated sodium channels, which prolongs depolarization and disrupts the generation and conduction of action potentials, especially in cardiac and neural cells [10]. This pathophysiological process underlies cardiac arrhythmias and central nervous system hyperexcitability, such as seizures, frequently seen in poisoning cases.

The present patient developed Life-threatening arrhythmias and profound hypotension, consistent with previous reports indicating that approximately 5.5% of hospitalized patients with aconitine poisoning die, with refractory arrhythmias and cardiac arrest being the major causes [8]. A review of recent case reports (Table 4) shows that such presentations are common across age groups and often involve severe outcomes, such as cardiac arrest, shock, and even death, despite aggressive interventions.. Sustained depolarization in cardiac cells has been consistently reported, leading to QT prolongation, impaired repolarization, and increased arrhythmogenic risk. In addition, aconitine's excessive activation of vagal nerve activity can cause sinus bradycardia and disrupt the autonomic balance, thereby increasing the risk of arrhythmias and hypotension [8]. Aconitine also induces vasodilation of vascular smooth muscle, decreasing peripheral resistance and contributing to hypotension, which may progress to shock when compounded by impaired cardiac function. The electrocardiographic manifestations in our patient, such as QT prolongation and ventricular arrhythmias, align with prior clinical and experimental observations [10].

**Table 4:** Literature review summary

Journal	Authors	PMID	Years	Case presentation	Diagnosis	Treatment	Outcome
World J Clin Cases	Liao YP, Shen LH, Cai LH, Chen J, Shao HQ	36,483,800	2022	61-year-old male, severe arrhythmia and cardiogenic shock caused by aconitine poisoning	Aconitine poisoning	Advanced life support, heart transplantation	Recovered after heart transplantation
J Forensic Sci	Huang R, Pang Q, Zheng L, Duan R, Wang Y, Wang Z, Wang T	39,233,350	2024	Male, 50 mL homemade medicinal liquor ingestion leading to cyanosis, arrhythmia, and organ damage	Aconitine poisoning	Toxicological analysis, postmortem examination	Forensic investigation completed
J Forensic Sci	Ya Y, Zhixiang Z, Chao L, Wei Z, Zhiyong W, Huafeng C, Shaohua Z, Hongfei X	34,235,734	2021	65-year-old man, accidental death after ingesting Chinese medicinal wine containing aconitine	Aconitine poisoning	LC–MS/MS analysis, supportive treatment	Death by aconitine poisoning
J Nippon Med Sch	Chen X, Wu R, Jin H, Gao R, Yang B, Wang Q	26,568,394	2015	48-year-old male, ingestion of herbal medicinal wine, arrhythmia, and polycystic renal hemorrhage	Aconitine poisoning, arrhythmia	Blood purification, heparin treatment	Recovery with polycystic kidney hemorrhage resolution
J Med Case Rep	Mjølstad OC, Radtke M, Brodtkorb E, Edvardsen F, Brede WR, Aamo TO, Jacobsen D, Stokke MK, Helland A	38,129,927	2023	53-year-old male with recurrent cardiac arrest and neurological symptoms, later diagnosed with aconitine poisoning	Aconitine poisoning	Multidisciplinary treatment, hair analysis confirmation	Full recovery, no further episodes
Hum Exp Toxicol	Lin CC, Phua DH, Deng JF, Yang CC	20,937,638	2011	60-year-old male, chest tightness, elevated troponin I, and abnormal scintigraphy following aconitine ingestion	Aconitine intoxication	Supportive management	Recovery
Perfusion	Zhao Z, Fang Z	39,196,956	2024	Patient in shock, treated with ECMO and hemoperfusion after aconitine poisoning	Aconitine poisoning, shock	ECMO, hemoperfusion, anti-shock therapy	Full recovery after ECMO support
Clin Case Rep	Bonanno G, Ippolito M, Moscarelli A, Misseri G, Caradonna R, Accurso G, Cortegiani A, Giarratano A	32,274,038	2020	Accidental aconitine poisoning, presenting with ventricular tachycardia and cardiac arrest	Aconitine poisoning	Supportive care, no further details	Death
J Pharm Biomed Anal	Huang YF, He F, Cui H, Zhang YY, Yang HY, Liang ZS, Dai W, Cheng CS, Xie Y, Liu L, Liu ZQ, Zhou H	34,814,080	2022	Investigation of four toxic Aconitum alkaloids and their acute toxicity	Toxicological analysis	Animal model, systematic investigation	Understanding toxicity
Drug Test Anal	Bicker W, Monticelli F, Bauer A, Roider G, Keller T	23,749,589	2013	3 cases of suicidal Aconitum napellus poisoning	Aconitine poisoning	Supportive care, including cardiopulmonary resuscitation and anti-arrhythmic drugs	Fatal; Aconitine as the cause of death
Forensic Sci Med Pathol	Cho YS, Choi HW, Chun BJ, Moon JM, Na JY	31,802,365	2020	81-year-old woman ingested liquid from Aconitum root, presented with ventricular arrhythmias and cardiac arrest	Aconitine poisoning	CPR, anti-arrhythmic drugs	Fatal; Aconitine levels quantified in body fluids

Currently, no specific antidote exists for aconitine poisoning. Symptomatic support, reduction of toxin absorption, enhancement of elimination, and blood purification techniques such as hemoperfusion combined with continuous renal replacement therapy (CRRT) have been frequently utilized, especially in severe cases [12, 15, 16]. Early initiation of these measures may accelerate toxin clearance and prevent complications such as acute kidney injury. Antiarrhythmic agents, notably amiodarone and lidocaine, are frequently used to manage refractory ventricular arrhythmias, with some reports suggesting that mexiletine may be more effective than lidocaine, though its use remains limited [17, 18]. Combination therapy is generally more effective than monotherapy [10]. Electrical cardioversion and defibrillation remain vital for managing life-threatening arrhythmias, though restoration of sinus rhythm is not always achieved [9, 19]. In this patient, a transient pacemaker was not applied. The episodes of ventricular tachycardia were short-lasting and spontaneously terminated, and hemodynamic stability was restored after norepinephrine infusion, amiodarone administration, and blood purification therapy. Therefore, the multidisciplinary team decided not to insert a pacemaker. Nevertheless, transient pacing has been reported as an effective acute intervention in drug-induced QT prolongation complicated by recurrent torsade de pointes or bradyarrhythmia, and it remains a valuable therapeutic option in refractory cases [20].

In recent years, additional therapies such as ginseng have been proposed to enhance detoxification, although the underlying mechanisms remain elucidated [21]. In cases of cardiac arrest unresponsive to standard interventions, prolonged cardiopulmonary resuscitation and extracorporeal life support (ECLS) such as ECMO may be lifesaving [22, 23]. VA-ECMO is particularly recommended for patients with severe circulatory collapse, because it provides respiratory and hemodynamic support. Aconitine is primarily eliminated through renal metabolism, and hypotension may further reduce renal perfusion, leading to acute kidney injury and metabolic acidosis [5, 24]. Elevated lactate and worsening acidosis can create a vicious cycle, aggravating organ dysfunction. Central nervous system depression, often exacerbated by acidosis, may progress from confusion and somnolence to coma, underscoring the importance of prompt recognition and correction of metabolic derangements.

Our patient benefitted from early multidisciplinary interventions, including blood purification, antiarrhythmic therapy, and supportive care, resulting in the resolution of acute symptoms and restoration of normal cardiac function. At 6 months of follow-up, the patient remained free of arrhythmia and exhibited stable cardiac performance. This favorable outcome demonstrates the potential effectiveness of an integrated treatment strategy, consistent with literature advocating for early, comprehensive management of severe aconitine poisoning. Nevertheless, timely diagnosis remains a challenge, and the effectiveness of different interventions requires validation through larger multicenter studies.

This case demonstrates that although multimodal treatment, including gastrointestinal decontamination, pharmacological intervention, blood purification, and cardiac pacing, can delay disease progression somewhat, rapid toxin distribution and irreversible organ damage may still limit therapeutic efficacy. Early in poisoning, aconitine can quickly affect the heart and nervous system, leading to severe arrhythmias, hypotension, and multiple organ dysfunction. To improve patient outcomes, future management should focus on early diagnosis and individualized intervention strategies, such as sensitive toxicological testing, specific antidotes, sodium channel blockers, and timely mechanical support. It should be noted that this report is based on a single case without a control group, and the limited sample size cannot fully reflect the clinical heterogeneity of aconitine poisoning or the generalizability of various treatment approaches. The effectiveness and safety of these interventions require validation in larger, multicenter prospective studies, and the underlying mechanisms and long-term outcomes warrant further investigation.

## Conclusion

This study presents a case of acute aconitine poisoning and systematically reviews the pathophysiological mechanisms, clinical features, and comprehensive treatment strategies, including pharmacological therapy, blood purification, and electrical cardioversion. The findings emphasize the importance of early recognition and intervention, and demonstrate that a multimodal approach can significantly improve patient prognosis. Electrocardiographic abnormalities are highlighted as early diagnostic markers, supporting the development of standardized diagnostic and therapeutic pathways for aconitine poisoning (Graphic abstract).

However, as this report is based on a single case, the results may not fully reflect the clinical heterogeneity of aconitine poisoning, and the treatment protocol has not been validated in randomized controlled trials. Further research with larger, multicenter studies is necessary to confirm the effectiveness and safety of these strategies. In addition, in-depth investigation into the molecular mechanisms of aconitine toxicity will help identify precise diagnostic biomarkers and novel therapeutic targets, ultimately optimizing the clinical management of toxicological emergencies.

## Data Availability

All data can be provided as needed.

## Abbreviations

AKI: Acute kidney injury
BE: Base excess
CRRT: Continuous renal replacement therapy
CNS: Central nervous system
CPR: Cardiopulmonary resuscitation
CVVH: Continuous venovenous hemofiltration
ECG: Electrocardiogram
ECLS: Extracorporeal life support
ECMO: Extracorporeal membrane oxygenation
EICU: Emergency Intensive Care Unit
HP: Hemoperfusion
LVEF: Left ventricular ejection fraction
PCB: Percutaneous cardiopulmonary bypass
VV-ECMO: Venovenous extracorporeal membrane oxygenation
VA-ECMO: Venoarterial extracorporeal membrane oxygenation
GCS: Glasgow Coma Scale

## References

[1] Liang X, Su W, Zhang W, et al. An overview of the research progress on *Aconitum carmichaelii* Debx.: active compounds, pharmacology, toxicity, detoxification, and applications. J Ethnopharmacol. 2025;337:118832. 10.1016/j.jep.2024.118832. 39306209 10.1016/j.jep.2024.118832

[2] Chan TY. Causes and prevention of herb-induced aconite poisonings in Asia. Hum Exp Toxicol. 2011;30(12):2023–6. 10.1177/0960327111407224. 21508072 10.1177/0960327111407224

[3] Jiang H, Li X, Fan Y, et al. The acute toxic effect of Chinese medicine Fuzi is exacerbated in kidney yang deficiency mice due to metabolic difference. J Ethnopharmacol. 2024;328:118036. 10.1016/j.jep.2024.118036. 38460575 10.1016/j.jep.2024.118036

[4] Lin X, Zhang J, Wu Z, et al. Involvement of autophagy in mesaconitine-induced neurotoxicity in HT22 cells revealed through integrated transcriptomic, proteomic, and M6A epitranscriptomic profiling. Front Pharmacol. 2024. 10.3389/fphar.2024.1393717. 38939838 10.3389/fphar.2024.1393717 PMC11208636

[5] Mi L, Li Y-C, Sun M-R, et al. A systematic review of pharmacological activities, toxicological mechanisms and pharmacokinetic studies on aconitum alkaloids. Chin J Nat Med. 2021;19(7):505–20. 10.1016/s1875-5364(21)60050-x. 34247774 10.1016/S1875-5364(21)60050-X

[6] Li L, Zhang L, Liao T, et al. Advances on pharmacology and toxicology of aconitine. Fundamemntal Clinical Pharma. 2022;36(4):601–11. 10.1111/fcp.12761. 10.1111/fcp.12761 35060168

[7] Chan T. Aconitum alkaloid poisoning related to the culinary uses of aconite roots. Toxins. 2014;6(9):2605–11. 10.3390/toxins6092605. 25184557 10.3390/toxins6092605 PMC4179150

[8] Chan TYK. Aconite poisoning. Clin Toxicol. 2009;47(4):279–85. 10.1080/15563650902904407. 10.1080/15563650902904407 19514874

[9] Lawson C, McCabe DJ, Feldman R. A narrative review of aconite poisoning and management. J Intensive Care Med. 2024;40(8):811–7. 10.1177/08850666241245703. 38613376 10.1177/08850666241245703

[10] Coulson JM, Caparrotta TM, Thompson JP. The management of ventricular dysrhythmia in aconite poisoning. Clin Toxicol. 2017;55(5):313–21. 10.1080/15563650.2017.1291944. 10.1080/15563650.2017.1291944 28421842

[11] Chan TYK. Aconitum alkaloid content and the high toxicity of aconite tincture. Forensic Sci Int. 2012;222(1–3):1–3. 10.1016/j.forsciint.2012.02.026. 22469654 10.1016/j.forsciint.2012.02.026

[12] Wu J, Lu AD, Zhang LP, Zuo YX, Jia YP. Zhonghua Xue Ye Xue. Za Zhi. 2019;40(1):52–7. 10.3760/cma.j.issn.0253-2727.2019.01.010. 10.3760/cma.j.issn.0253-2727.2019.01.010 PMC7351698 30704229

[13] Tai C-J, El-Shazly M, Wu T-Y, et al. Clinical aspects of Aconitum preparations. Planta Med. 2015;81(12/13):1017–28. 10.1055/s-0035-1546183. 26166138 10.1055/s-0035-1546183

[14] Majumder MI, Mahadi AR, Rahman OU, et al. Accidental poisoning with aconite overdose: a case report and resuscitative emergency management in a tertiary level hospital of Bangladesh. Clin Case Rep. 2023. 10.1002/ccr3.7845. 37636875 10.1002/ccr3.7845 PMC10448233

[15] Baylis S, Costa-Pinto R, Hodgson S, et al. Combined hemoperfusion and continuous veno-venous hemofiltration for carbamazepine intoxication. Blood Purif. 2021;51(9):721–5. 10.1159/000520520. 34879379 10.1159/000520520

[16] Zhang M, Zhang W, Zhao S, et al. Hemoperfusion in combination with hemofiltration for acute severe organophosphorus pesticide poisoning. J Res Med Sci. 2022;27(1):33. 10.4103/jrms.jrms_822_20. 35548179 10.4103/jrms.JRMS_822_20 PMC9081518

[17] Gottignies P, El Hor T, Tameze JK, et al. Successful treatment of monkshood (*Aconite napel*) poisoning with magnesium sulfate. Am J Emerg Med. 2009;27(6):755.e1-755.e4. 10.1016/j.ajem.2008.10.008. 19751645 10.1016/j.ajem.2008.10.008

[18] Lin C-C, Chan TYK, Deng J-F. Clinical features and management of herb-induced aconitine poisoning. Ann Emerg Med. 2004;43(5):574–9. 10.1016/j.annemergmed.2003.10.046. 15111916 10.1016/j.annemergmed.2003.10.046

[19] Michel A, Siebe I, Auwärter V, et al. Aconitinvergiftung Durch Eine Verwechslung von Eisenhutblättern Mit Liebstöckel. Anaesthesist. 2021;70(8):633–8. 10.1007/s00101-021-01002-w. 34251482 10.1007/s00101-021-01002-w

[20] Viskin S, Glikson M, Fish R, et al. Rate smoothing with cardiac pacing for preventing Torsade de Pointes. Am J Cardiol. 2000;86(9):K111–5. 10.1016/s0002-9149(00)01228-5. 10.1016/s0002-9149(00)01228-5 11084109

[21] Bao Y, Zhang R, Jiang X, et al. Detoxification mechanisms of ginseng to aconite: a review. J Ethnopharmacol. 2023;304:116009. 10.1016/j.jep.2022.116009. 36516908 10.1016/j.jep.2022.116009

[22] Lorusso R, Shekar K, MacLaren G, et al. ELSO interim guidelines for venoarterial extracorporeal membrane oxygenation in adult cardiac patients. ASAIO J. 2021;67(8):827–44. 10.1097/mat.0000000000001510. 34339398 10.1097/MAT.0000000000001510

[23] Kohara S, Kamijo Y, Kyan R, et al. Severe aconite poisoning successfully treated with veno-arterial extracorporeal membrane oxygenation: a case report. World J Clin Cases. 2024;12(2):399–404. 10.12998/wjcc.v12.i2.399. 38313648 10.12998/wjcc.v12.i2.399 PMC10835702

[24] Fujita Y, Terui K, Fujita M, et al. Five cases of aconite poisoning: toxicokinetics of aconitines. J Anal Toxicol. 2007;31(3):132–7. 10.1093/jat/31.3.132. 17579959 10.1093/jat/31.3.132

